# Emergency Care Systems: The Missing Link for Effective Treatment of COVID-19 in Africa

**DOI:** 10.1017/dmp.2020.239

**Published:** 2020-07-14

**Authors:** Emilie J. Calvello Hynes, Corey B. Bills

**Affiliations:** Department of Emergency Medicine, University of Colorado School of Medicine, Aurora, CO

**Keywords:** disease outbreaks, emergency medicine, emergency service, health systems, hospital

## Abstract

Cases of COVID-19 are rising quickly on the African continent. A critical element of any health system response to such a surge of active cases is the existence of functional emergency care systems. Yet, these systems are markedly underdeveloped in African countries. This short letter reviews the key role emergency medicine plays in epidemic disease response and actions that ministries of health can take now to shore up gaps in emergency care capacity to avoid needless death and suffering of COVID-19 patients.

The rising spread of severe acute respiratory syndrome coronavirus 2 (SARS-CoV-2) on the African continent is gravely concerning. In recent weeks, there has been a large rise in documented coronavirus disease (COVID-19) cases, with over 1 million current cases and 23 000 deaths.^1^ The United Nations estimates the disease will cause at least 300 000 deaths in Africa and shift another 30 million people into poverty.^2^

During the current pandemic, emergency departments have been highlighted as mission critical locations to screen for syndromic disease, isolate and protect patients and health care workers, triage, and provide immediate care for emergency conditions associated with COVID-19, such as respiratory failure and shock. While emergency care systems are necessary for a successful health sector response, they remain inadequately supported in low- and middle-income countries. A review of 59 countries highlighted the major limitations of emergency care delivery: markedly higher mortality rates than high-income countries and inadequate training across all cadres of health care providers.^3^

During epidemics, weak emergency care systems can become overwhelmed by increased demand or directly compromised by the impact of the outbreak. When service delivery is undermined, both direct disease mortality and preventable mortality from everyday emergency conditions can increase dramatically. This is especially true of emergent health conditions that rely on skilled health personnel, medicine, and equipment for treatment. During the 2014 Ebola outbreak, strains on the overall health system led to excess mortality from non-Ebola-related conditions, including malaria and emergency obstetric conditions.^4^

Targeted capacity augmentation for emergency departments is necessary now to avoid excess mortality from the expected surge in COVID-19 cases in Africa. Table 1 recommends targeted interventions, based on guidance provided in the 2019 World Health Assembly Resolution 72.16, “Emergency care systems for universal health coverage: ensuring timely care for the acutely ill and injured,” that can have far reaching implications for health outcomes.^5^

**TABLE 1:** Targeted Interventions to Increase Capacity in African Emergency Departments for Expected COVID-19 Surge

**Screening:** Targeted training of health care workers to identify and isolate those meeting the COVID-19 case definition when presenting to emergency departments.
**Triage:** Resource appropriate triage implementation with processes to redirect possible COVID-19 patients away from the primary intake holding area.
**Critical supplies:** Augment personal protective equipment and essential equipment to manage acute hypoxic respiratory failure (eg, pulse oximeter, oxygen delivery equipment, oxygen supply, masks, inhalers).
**Case management guidance for resource limited settings:** Standardize case management for acute hypoxic respiratory failure associated with COVID-19 and other critical conditions.
**Out-of-hospital care:** Promote access to prehospital care and establish criteria for both prehospital and interfacility transport of critically ill patients to designated care centers.
**Crisis Standards of Care:** Develop tiered, proactive strategies to optimize resources that emphasize adapting staff and supply shortages for the COVID-19 response.
**Palliative care:** Provide clear clinical guidance about the compassionate use of palliative care for COVID-19 patients.

As Africa braces for a possible explosion of COVID-19 cases in the coming months, excess mortality is not inevitable. Timely, strategic implementation of targeted emergency care solutions in African countries can help avoid needless human suffering and death.
